# Microbial diversity of co-occurring heterotrophs in cultures of marine picocyanobacteria

**DOI:** 10.1186/s40793-020-00370-x

**Published:** 2021-01-06

**Authors:** Sean M. Kearney, Elaina Thomas, Allison Coe, Sallie W. Chisholm

**Affiliations:** Department of Civil and Environmental Engineering, Massachusetts Institute of Technology, 15 Vassar St, Cambridge, MA 02139 USA

**Keywords:** *Prochlorococcus*, *Synechococcus*, Phycosphere, 16S, Copiotrophs

## Abstract

**Background:**

The cyanobacteria *Prochlorococcus* and *Synechococcus* are responsible for around 10% of global net primary productivity, serving as part of the foundation of marine food webs. Heterotrophic bacteria are often co-isolated with these picocyanobacteria in seawater enrichment cultures that contain no added organic carbon; heterotrophs grow on organic carbon supplied by the photolithoautotrophs. For examining the selective pressures shaping autotroph/heterotroph interactions, we have made use of unialgal enrichment cultures of *Prochlorococcus* and *Synechococcus* maintained for hundreds to thousands of generations in the lab. We examine the diversity of heterotrophs in 74 enrichment cultures of these picocyanobacteria obtained from diverse areas of the global oceans.

**Results:**

Heterotroph community composition differed between clades and ecotypes of the autotrophic ‘hosts’ but there was significant overlap in heterotroph community composition across these cultures. Collectively, the cultures were comprised of many shared taxa, even at the genus level. Yet, observed differences in community composition were associated with time since isolation, location, depth, and methods of isolation. The majority of heterotrophs in the cultures are rare in the global ocean, but enrichment conditions favor the opportunistic outgrowth of these rare bacteria. However, we found a few examples, such as bacteria in the family Rhodobacteraceae, of heterotrophs that were ubiquitous and abundant in cultures and in the global oceans. We found their abundance in the wild is also positively correlated with that of picocyanobacteria.

**Conclusions:**

Particular conditions surrounding isolation have a persistent effect on long-term culture composition, likely from bottlenecking and selection that happen during the early stages of enrichment for the picocyanobacteria. We highlight the potential for examining ecologically relevant relationships by identifying patterns of distribution of culture-enriched organisms in the global oceans.

## Background

Marine microorganisms perform matter and energy transformations that underlie global biogeochemical cycles. Fixation of carbon dioxide by autotrophs forms the base of these transformations. The picocyanobacteria *Prochlorococcus* and *Synechococcus* contribute approximately 25% of this global ocean net primary productivity [1].

Abundant, free-living oligotrophic bacteria like *Prochlorococcus,* many marine *Synechococcus,* and heterotrophs in the SAR11 clade exhibit genome streamlining, likely driven by evolutionary pressures on bacteria with large population sizes in relatively stable environments [2–4]. These three groups together often represent more than 50% of free-living bacterial cells in the oligotrophic surface oceans [5, 6]. By contrast, marine copiotrophs typically have larger genomes and cell sizes, and display periodic fluctuations in abundance, depending on influx of organic matter [7–10].

Unlike the low nutrient regimes of the oligotrophic oceans, cultures of *Prochlorococcus* and *Synechococcus* have high concentrations of inorganic nutrients (N, P, Fe) and accumulate dissolved organic matter and detritus [11, 12]. These conditions likely favor heterotrophic microorganisms adapted to higher organic carbon concentrations such as those associated with detrital particles or phytoplankton blooms [8, 13–16]. Even in oligotrophic oceans, concentrations of organic matter can be elevated in the diffusive boundary layer surrounding phytoplankton cells – the “phycosphere” [14, 17]. A phycosphere’s size is a function of cell size, thus it can extend hundreds to thousands of microns from the surface of large eukaryotic phytoplankton; that of *Prochlorococcus* and *Synechococcus* span only a few cell diameters– i.e. 1–2 μm [14]. Assembly of heterotrophic bacterioplankton in the phycosphere depends on the nature of diverse organic compounds exuded [12, 18, 19], and these heterotrophs can alter the physiology of their primary producer hosts [20, 21].

Many studies demonstrate interactions between bacterioplankton and eukaryotic hosts in the phycosphere, but heterotrophs also exhibit chemotaxis towards metabolites derived from *Prochlorococcus* and *Synechococcus* [22]. Rather than deriving dissolved organic carbon (DOC) from relatively limited diffusive boundary layers around picocyanobacteria, they most likely obtain this carbon by free diffusion in bulk seawater [23]. There’s also evidence, however, that heterotrophs might attach directly to a picocyanobacterial host [24] or associate with them while attached to particulate matter [25], which would be proximal enough to experience a phycosphere.

Cyanobacterial cultures accumulate cells and organic carbon, leading to physical interactions and metabolic exchanges between the phototroph and associated heterotrophs. Indeed, *Prochlorococcus* in culture can exhibit either growth improvement or inhibition when co-cultured with various microbes [26–28]. Co-culture benefits to cyanobacteria include improved recovery from, and longevity in, stationary phase [29], revival from low cell numbers [30], tolerance to temperature changes [31], and survival under extended darkness [32, 33]. However, co-culture can also delay the onset of growth in some strains of *Prochlorococcus* [26], and high densities of otherwise beneficial strains can cause inhibition [27].

When interactions are beneficial, heterotrophic bacteria often provide complementary functions for their “host” cyanobacteria, such as the production of catalase to detoxify reactive oxygen species [30, 34, 35], and the recycling and release of reduced N [36, 37] and P [38]. In some cases, this nutrient recycling fosters indefinite longevity [38].

Because of beneficial interdependencies, separating cyanobacteria from heterotrophic “helpers” (sensu Morris et al. 2008) in enrichment cultures presents a challenge for obtaining axenicity [30, 38]. However, the enrichment cultures present a unique opportunity to investigate selective effects on self-assembled mixed microbial communities. While the boom-bust and high nutrient conditions of batch culture do not mimic the conditions present in the open ocean (except perhaps on particles or in phytoplankton blooms), they effectively extend the phycosphere throughout the culture such that we can observe forces acting on wild communities. The observations can inform future work on the dynamics of particle-attachment or aggregation among picocyanobacteria and associated heterotrophs, and provide insights for obtaining and sustaining axenic strains of picocyanobacteria.

We have maintained cultures of *Prochlorococcus* (56) and *Synechococcus* (18), and accompanying heterotrophic “bycatch” for hundreds to thousands of generations in the laboratory by serial liquid transfer, and have used this collection to address several questions about the nature of these phototroph-associated microbial communities: What is the composition and diversity of these heterotroph communities after many passages? How does the composition of our long-term cultures compare to that in cultures of picocyanobacteria or diatoms maintained for shorter periods? Which factors determine composition of the heterotroph community: the method of isolation, the age of the culture, the specific time and place of isolation? And finally, how does the composition of heterotrophs in culture compare to the communities in the waters from which the host cyanobacterium was isolated?

## Results and discussion

### The cyanobacterial culture collection

**Fig. 1:**
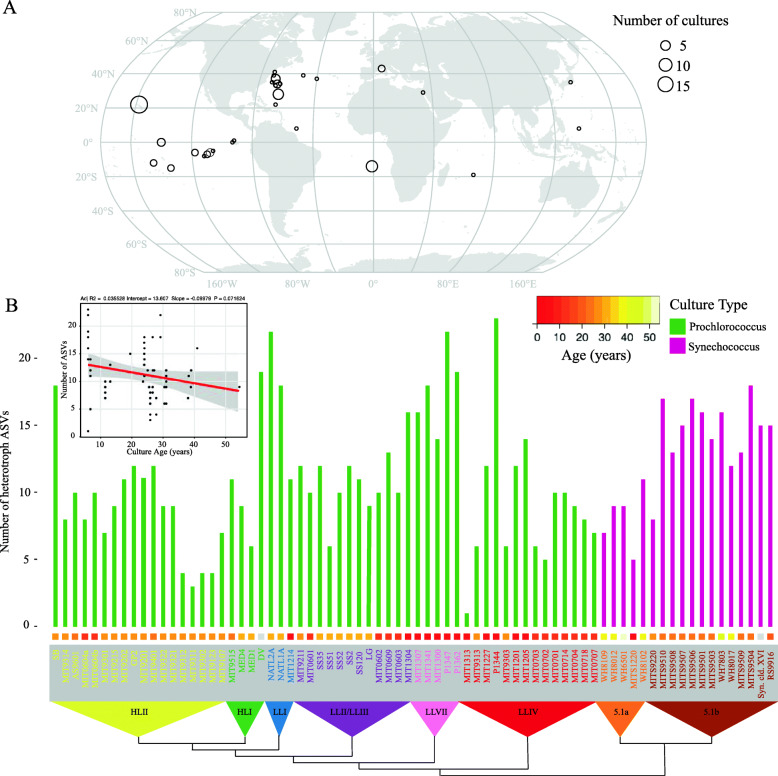
Microbial diversity in enrichment cultures from the global oceans. **a** Origin of *Prochlorococccus* and *Synechococcus* cultures obtained over several decades and maintained in serial transfer batch cultures for 100 s – 1000s of generations. The size of each point on the map represents the number of cultures obtained from a given location. For date and methods of isolation see Table S1. **b** Richness of heterotrophic bacteria measured by the number of amplicon sequence variants (ASVs) exceeding a threshold relative abundance of 0.2% in *Prochlorococcus* (green) and *Synechococcus* (magenta) cultures. Richness is organized by the phylogeny of host organisms (built using the 16S–23S intertranscribed spacer (ITS) sequence, and collapsed to indicate monophyletic groups) indicated along the bottom. HL (high light) and LL (low light) designations in the triangles refer to light-adaptation features of *Prochlorococcus* ecotypes, as reviewed in (Biller et al. 2014 [39]), and 5.1a and 5.1b refer to subclusters of *Synechococcus* group 5.1 (Ahlgren and Rocap [40]). Colored boxes above each culture name indicate the years between when the culture was isolated and this analysis, as specified by the color bar in the upper left-hand corner. Inset: regression between culture age and richness

The cyanobacterial isolates (co-isolated with unknown consortia of heterotrophs, the subjects of this investigation) used in the analysis span multiple clades of *Prochlorococcus* and *Synechococcus* obtained from many locations and times (between 1965 and 2013) in the global oceans (Fig. 1a, Table S1). The isolates – most of which are from the oligotrophic oceans – have been maintained by serial transfer for many years, with cultures ranging in age from 6 to 55 years, and thus represent what we believe to be stable consortia of single algal isolates associated with diverse heterotrophic bacterial communities (see Materials and Methods, and Figure S1) (Fig. 1b).

### Composition and diversity of heterotroph communities in the cultures

**Fig. 2:**
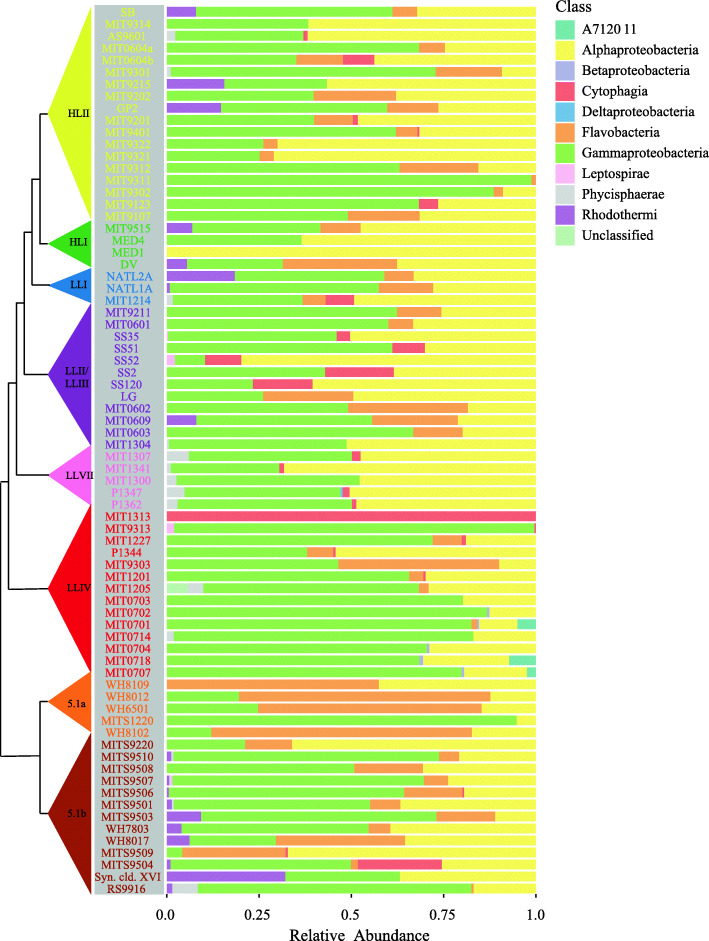
Composition of heterotroph communities, as defined by class membership of ASVs, in long-term cultures of *Prochlorococcus* and *Synechococcus* hosts (tree on the left as defined in Fig. 1). The heterotroph communities in each culture are arranged using the phylogenetic tree of the host cyanobacterium based on the ITS sequence and collapsed into monophyletic groups as in Fig. 1b. Relative abundance of heterotrophic community members, by class, is shown to the right of each strain name. To view the same data by hierarchical clustering see Figure S2.

We anticipated that heterotrophic culture richness – as defined by amplicon sequence variants (ASVs) of the V4 region of 16S rRNA gene – might decrease with age of the culture due to extinctions over time. However, the number of ASVs, which ranged from 1 to 23 (Fig. 1b), was only weakly and not significantly anti-correlated with the age of the culture (Spearman’s rho = − 0.19, *p*-value = 0.11). NATL2A, for example, at nearly 30 years old, is one of the oldest cultures but has 22 ASVs, while P1344 is 6 years old and contains 23 ASVs – a difference of just one ASV for an age difference of 24 years. Further, we sampled three sets of cultures (*Prochlorococcus* str. MED4, NATL2A, and MIT9313) in 2018 and 1 year later, and found a general correspondence in the community composition over time (Figure S1). Cultures of the same *Prochlorococcus* maintained by different individuals (MIT0604a & MIT0604b) also showed similar community composition, as well as cultures derived from the same starting enrichment culture (e.g. SS2, SS35, SS51, SS52, and SS120 were all derived from the LG culture (Table S1)) (Fig. 2). These results suggest that the heterotroph communities in these cultures remain similar over time and independent maintenance, but exhibit slight drift over time (i.e. the cultures are similar but not identical in composition) (Figure S1).

**Fig. 3:**
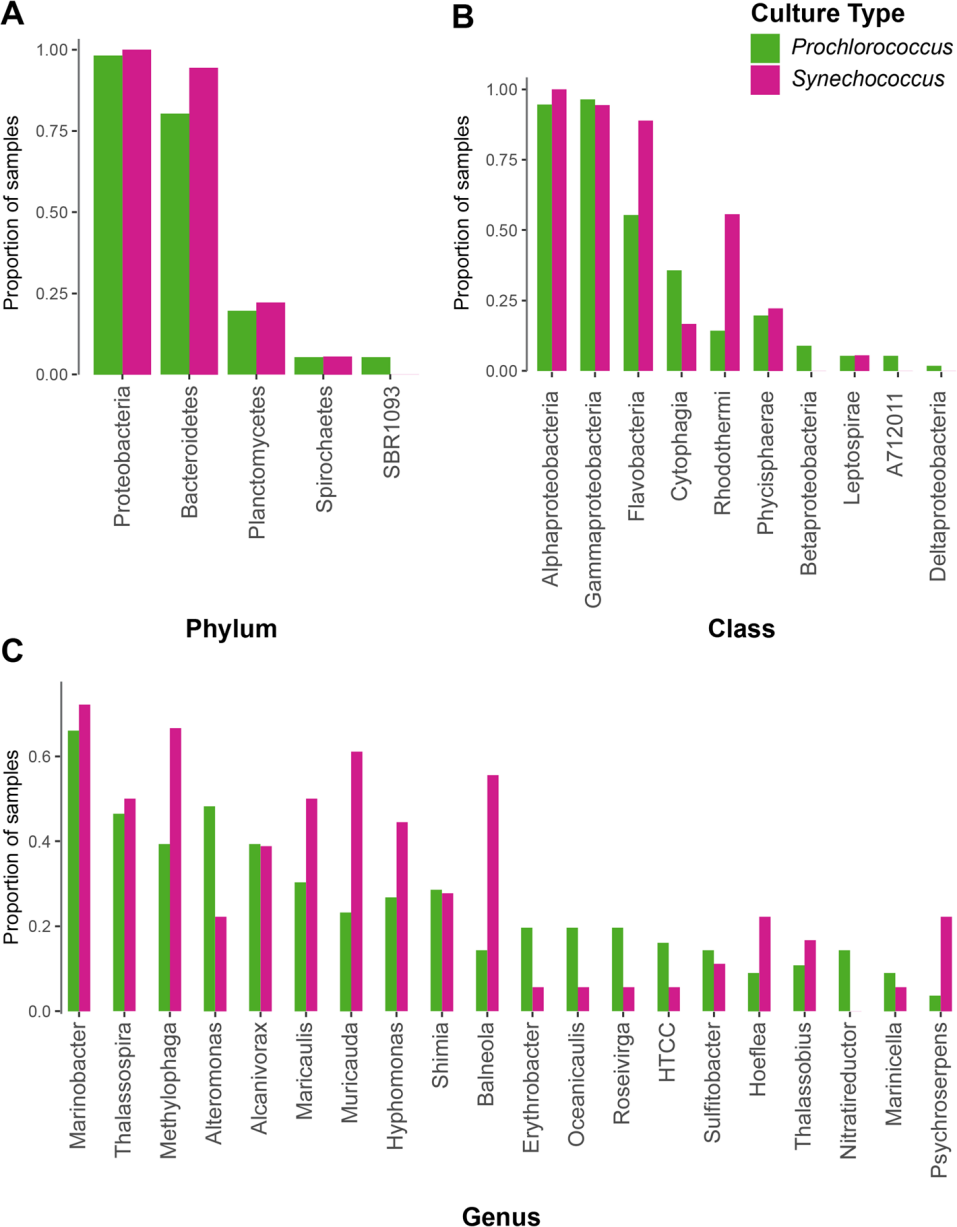
Proportion of cultures of either *Prochlorococcus* (green) or *Synechococcus* (magenta) containing ASVs belonging to a given bacterial (**a**) phylum, **b** class, or **c** genus (restricted to genera found in at least six cultures). Note that HTCC is listed in the Greengenes taxonomy, but is not formally recognized as a bacterial genus

We next explored whether the heterotroph community composition was related to the cyanobacterial “host” – i.e. *Prochlorococcus* or *Synechococcus –* in the cultures. Which taxonomic groups were common to both, and which were more common in one or the other? Grouping ASVs at the phylum level, heterotroph communities in the cultures were largely comprised of bacteria from the phyla Proteobacteria (primarily in the classes Alpha- and Gammaproteobacteria, with some Delta- and Betaproteobacteria), Bacteroidetes (classes Cytophagia, Flavobacteria, and Rhodothermi), and Planctomycetes (class Phycisphaerae), with a minor contribution from Spirochaetes (class Leptospirae) and candidate phylum SBR1093 (class A712011) (Fig. 2, Figure S2). SBR1093 was only in cultures of the LLIV clade of *Prochlorococcus* (Fig. 2). With respect to heterotrophic phyla, *Prochlorococcus* and *Synechococcus* cultures contain Proteobacteria and Planctomycetes at similar frequencies, but Bacteroidetes are more common, though not significantly (Fisher Exact Test *p*-values > 0.05), in *Synechococcus* cultures (present in 94% of cultures versus 80%) (Fig. 3a). At the class level, Alpha- and Gammaproteobacteria as well as Phycisphaerae have equal representation across the two cyanobacterial hosts (Fig. 3b). Flavobacteria (present in 90% of *Synechococcus* cultures versus 56% of *Prochlorococcus*, Fisher Exact Test *p*-value = 0.01) and Rhodothermi (present in 56% of *Synechococcus* cultures versus 14% of *Prochlorococcus*, Fisher Exact Test *p*-value = 0.06), however, are more well-represented in *Synechococcus* cultures, suggesting that compounds in the exudate derived from *Synechococcus* may be better matched to their growth requirements. Indeed, *Prochlorococcus* and *Synechococcus* secrete distinct compounds during growth [12], few of which are characterized, but likely promote differential growth of associated heterotrophs as shown previously [41, 42].

Minor differences in heterotroph communities between *Prochlorococcus* and *Synechococcus* hosts at the phylum and class level suggested that there might be more pronounced differences at the genus level. This was not the case, however; instead they were quite similar (no significant differences after multiple hypothesis correction) (Fig. 3c). For instance, the most ubiquitous genus (present in over 60% of both culture types) is *Marinobacter* (Fig. 3c), which is well-known to be associated with picocyanobacteria in culture [30]. In addition to *Marinobacter*, other genera present in at least 15% of cultures including *Thalassospira*, *Methylophaga*, *Alteromonas*, *Alcanivorax*, *Maricaulis, Muricauda*, and *Hyphomonas* (but not *Shimia*) have been associated with metabolism of hydrocarbons or C1 compounds derived from lipid catabolism [10, 43–54], suggesting that hydrocarbon metabolism might play an important role in their growth in these cultures. Indeed, previous work showed an upregulation of genes for fatty acid metabolism including lipid beta-oxidation in co-culture of *Alteromonas macleodii* with *Prochlorococcus* [28]. *Prochlorococcus* secretes vesicles (potentially a source of lipids) in culture and in the wild [39, 55], which can outnumber cells by a factor of 10 or more, and marine heterotrophs like *Alteromonas* are capable of growing on these vesicles as a sole source of carbon [39]. Recent work also suggests that *Alteromonas* isolates derived from cultures of *Prochlorococcus* carry genes for the degradation of aromatic compounds produced by the cyanobacterium [47], which may provide a selective advantage for *Alteromonas* in culture with *Prochlorococcus*. Additionally, metagenomics on a *Synechococcus*-associated culture revealed high abundance of TonB-dependent transporters in *Muricauda*, potentially involved in lipid uptake, and proteins involved in the export of lipids in the proteome of the *Synechococcus* host [42].

Finally, we investigated whether specific ASVs were differentially represented between culture types (*Prochlorococcus* versus *Synechococcus*). To identify taxa associated with a specific habitat (here either a *Synechococcus* or *Prochlorococcus* culture), we used indicator species analysis (see Materials and Methods), based on presence/absence data. We found that 20 ASVs were more prevalent among *Synechococcus* cultures than *Prochlorococcus* cultures, but only one ASV (a *Marinobacter* sequence) more prevalent in *Prochlorococcus* cultures (Table S2). These associations strengthen the possibility that the structure of heterotroph communities may arise in response to cyanobacterial host-specific secretion of compounds.

**Fig. 4:**
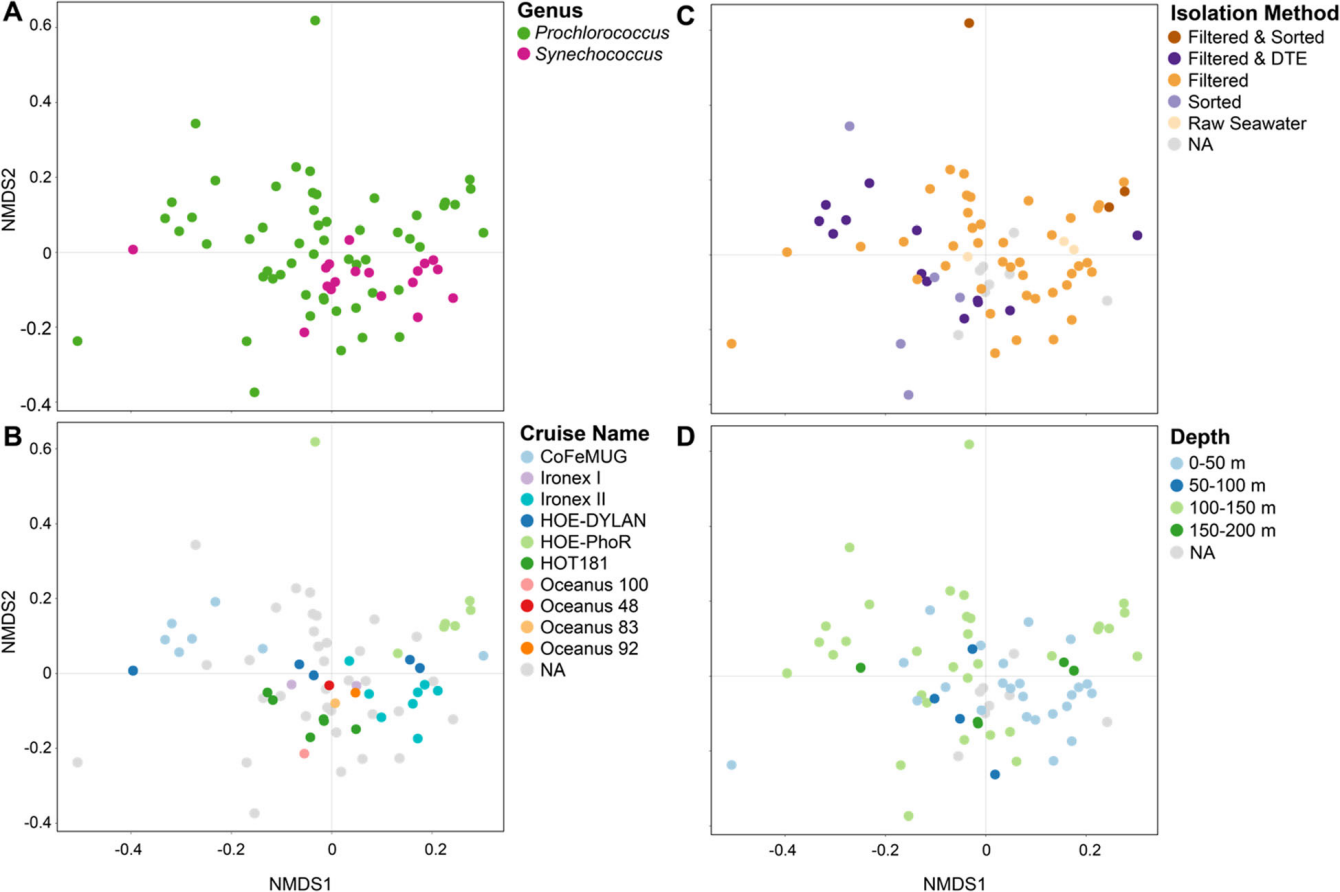
Ordination of heterotroph community composition at the ASV level overlaid with metadata pertaining to conditions of isolation of enrichment cultures. Non-metric multidimensional scaling (NMDS) of heterotroph communities using unweighted UniFrac as the distance metric overlaid with (**a**) the host cyanobacterium genus in the enrichment culture, **b** the cruise name from which the culture was isolated, **c** the isolation method (pre-filtered to exclude larger cells, pre-filtered and sorted via flow cytometry, pre-filtered and diluted-to-extinction (DTE), not filtered and sorted via flow cytometry, or cloned from a mother culture), and **d** the depth of the seawater sample. The closeness of two points in NMDS space reflects the distance between communities, with communities having more similar phylogenetic structure (as measured by unweighted UniFrac) grouping more closely together. Heterotroph communities for which metadata was not known are indicated as NAs. See also Figures S3, and S4 for relationships between community structure and ecotype, clade, isolation location, and culture age

Consistent with there being more indicator species in *Synechococcus* cultures, community composition was more similar across *Synechococcus* cultures than *Prochlorococcus* cultures (as measured by unweighted UniFrac distance at the ASV level (PERMANOVA, *p*-value < 0.01)) (Fig. 4a, Figures S3 and S4). Further, heterotroph community composition using the same metric was significantly associated (PERMANOVA, *p*-value < 0.01, Table S5) with cyanobacterial ecotype (Figure S4A) and clade (Figure S4C), but there were no discernible patterns in these associations.

### Comparison of composition with similar studies of diatoms and *Synechococcus*

To assess the similarity of other phytoplankton-associated microbiomes to those obtained in this study we re-processed – in the context of our dataset – 16S rDNA amplicon sequence data as 97% OTUs (see Materials and Methods) to limit noise and batch effects from differences in sample processing and sequencing approaches – from published diatom (Behringer et al. 2018 [56]) and recently isolated *Synechococcus*-associated microbial communities (Zheng et al. 2018 [41]). Surprisingly, the heterotroph communities in diatom and *Synechococcus* cultures each shared almost half the OTUs found in our cultures: 45% (13/29) for the diatoms and 41% (29/70) for the *Synechococcus* cultures (Table S3, Figure S5). Among these OTUs, 11 were present in cultures of all three groups of phytoplankton, potentially comprising a ‘core’ set of sequence clusters associated with phytoplankton isolates. These include three of the ubiquitous genera (*Roseivirga*, *Maricaulis*, and *Alteromonas*) from this study, which derive from three separate classes (Fig. 3c). At a high level, the taxonomic diversity in cultures from *A. tamarense* and *T. pseudonana* in a separate study (Fu et al. [57]) exhibited many of the same marine heterotrophs seen here (Figure S6), which suggests that diverse phytoplankton may have overlapping selective effects on their associated microbial communities.

In contrast to this core of shared heterotrophic bacteria, several additional classes (Acidimicrobiia, Actinobacteria, Planctomycetes class OM190, and Saprospirae) were represented in the Zheng et al. 2018 [41] *Synechococcus* dataset, but absent from our study; only the class Saprospirae was present in the diatom dataset and absent in ours (Figure S6). Notably, the Actinobacteria from the *Synechococcus* dataset were only found in isolates from eutrophic waters; by contrast, all except for two (*Prochlorococcus* SB and *Synechococcus* WH8017) of our isolates were from oligotrophic waters, while the diatoms of Behringer et al. 2018 [56] were isolated from coastal waters. Further work should examine the extent to which these differences are driven by starting inoculum versus physiological features of the host phytoplankton.

### Features of the sample of origin in determining community composition

We examined the extent to which differences in heterotroph communities – here measured at the level of ASVs – in the cultures were related to factors involved in isolation (Table S1). Specifically, we investigated whether the heterotroph composition between all pairs of communities varied with: cruise on which it was isolated, isolation method, sample location and depth, and date the culture was isolated. Each of the tested metadata variables showed a significant association (PERMANOVA, *p*-value < 0.01, Table S5) with differences in community composition as measured by unweighted UniFrac distance (a measure of the phylogenetic similarity between two communities), and there were no obvious cases in which the pattern of associations for one variable completely overlapped those for another (Fig. 4, Figure S3, Figure S4). Notably, richness (number of ASVs) in the cultures showed no association with any of the tested variables, as previously mentioned for culture age (Wilcoxon Rank Sum Test, *p*-value > 0.05). In other words, differences in the heterotroph membership of communities (unweighted UniFrac distance) associated with features of the sample of origin did not arise from differences in the total number of heterotroph ASVs (richness).

While “cruise” itself is not a particularly informative variable on its own, we did see a reproducible tendency for cultures obtained on the same cruise to have similar heterotroph community composition (PERMANOVA, *p*-value < 0.01, Table S5). For example, cultures with names beginning in MIT13 or P13 (i.e. MIT13*XX* or P13*X*)*,* were isolated by the same person on the same cruise (HOE-PhoR) in 2013 from a depth of 150 m, and include *Prochlorococcus* from multiple low light clades (LLIV, LLII/LLIII, and LLVII) (Table S1). They were isolated with a variety of methods including filtration, flow sorting, and dilution-to-extinction. With two exceptions (the LLIV cultures P1344 and MIT1313, the latter of which was dominated by a single heterotroph ASV after dilution-to-extinction) from the suite of eight cultures, the MIT13*XX* and P13*X* strains clustered together by unweighted UniFrac, suggesting a sensitive dependence of heterotroph community composition on the conditions – not solely the physical isolation methodology – under which the culture was first isolated (indeed, we see differences in beta-dispersion across several of the cruises, 10/39 pairwise comparisons with *p*-value < 0.05, 1/39 after Bonferroni correction) (Fig. 4b and Figure S2). These conditions include the initial composition of heterotrophic bacteria in the collected seawater sample, the specific media formulation or light and temperature conditions used for a given enrichment attempt, and the duration of time an enrichment culture was maintained before derivation of individual algal strains. Together, these factors may drive the observation that cultures obtained from a given cruise frequently share similar heterotroph communities (seen also with the CoFeMUG and EqPac/IRONEX cruises, whose beta-dispersion differed significantly, Bonferroni-adjusted *p*-value = 0.039) (Fig. 4b). We hypothesize that these similarities result from a bottlenecking of community diversity shortly after cultures are sampled and phytoplankton are enriched.

To look into the effects of potential bottlenecking during the initial culturing phase, we examined the relationship between culture composition and physical isolation methods (Fig. 4c). We see a tendency for cultures obtained using only filtration (not accompanied by dilution-to-extinction or flow sorting) to be more similar in heterotroph composition to each other than cultures also subjected to dilution-to-extinction or flow sorting (post-hoc tests on pairwise dispersion, Bonferroni adjusted *p*-values = 0.02) (Fig. 4c). These findings suggest that stochastic loss of heterotrophs that might occur by dilution-to-extinction reduces the structural convergence of heterotroph communities.

Finally, heterotroph community composition in the cultures differs by collection depth (Fig. 4d). For example, the heterotroph communities of cultures isolated from 0 to 50 m and 50–100 m tend to cluster more tightly with each other than cultures isolated from 100 to 150 m and 150–200 m, consistent with the idea that community composition of heterotrophs varies with depth (post-hoc test on pairwise dispersion between 100 and 150 m and 0–50 m, Bonferroni adjusted *p*-value = 0.006) [58–60]. This finding is not surprising given that light and nutrient gradients in the water column lead to more complex biogeochemical regimes – and hence a gradient in dissolved organic carbon compounds [61, 62] from the surface.

In conducting the above analyses, we note that some of these variables are not independent (e.g. because ecotype depends on clade; or because strains were only isolated from one depth or one ecotype was isolated on a given cruise), so associations may arise from interdependencies between factors (Figure S7, Table S5). These interdependencies may lead to associations with multiple variables which could be explained by a single variable. However, the overall trends show a clear linkage between initial conditions of culture isolation and the long-term composition of the culture.

### Comparison of cultured communities to distributions of their members in the wild

**Fig. 5:**
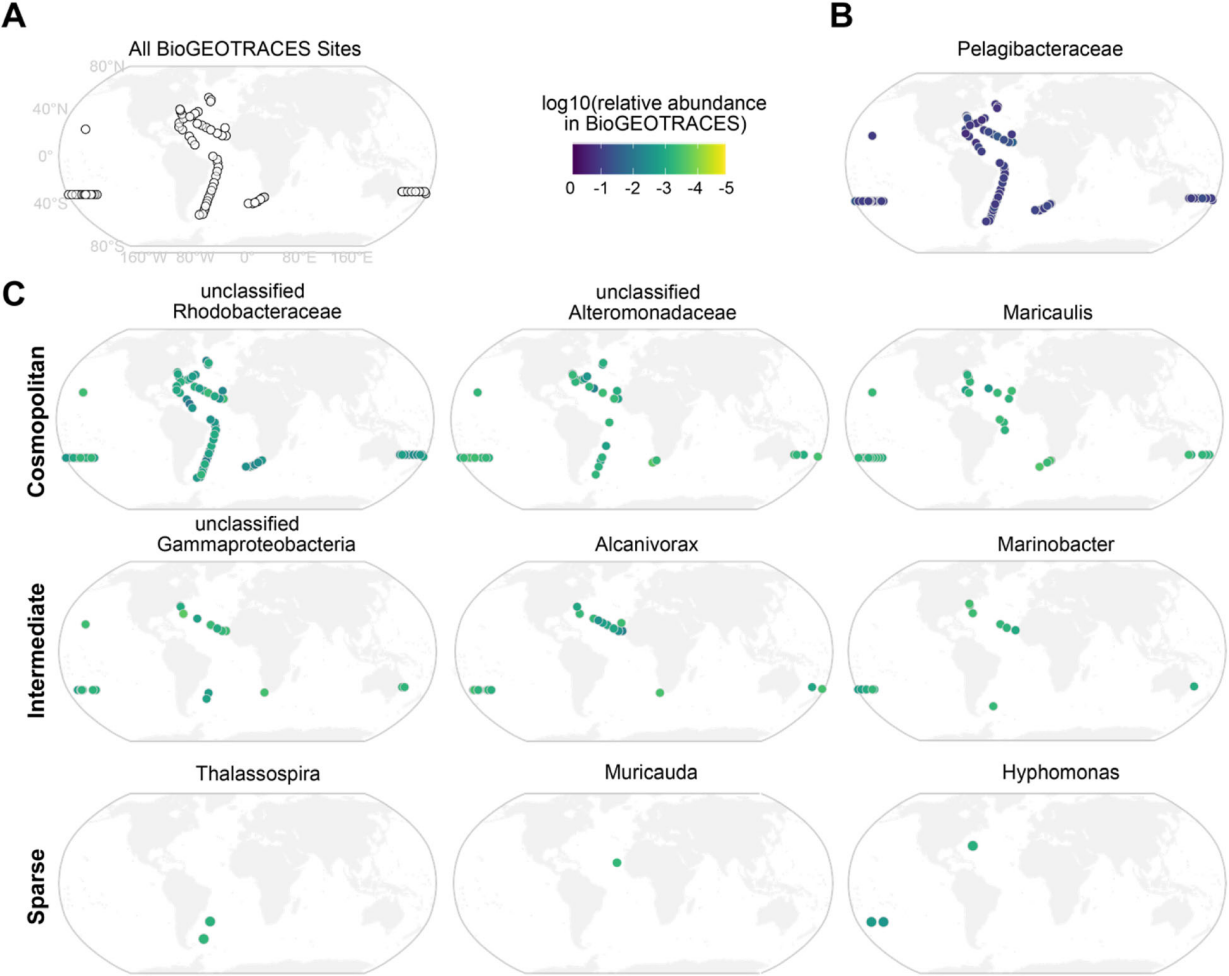
Representation of picocyanobacterial enrichment culture heterotrophic bacterial OTUs in global ocean surveys. **a** Location of the sampling sites of the bioGEOTRACES expeditions. **b** Example of a heterotrophic OTU (in the family Pelagibacteraceae) abundant in bioGEOTRACES, but absent from cultures. **c** Distribution of the most ubiquitous OTUs in cultures across the bioGEOTRACES sites. Such OTUs were present in almost all bioGEOTRACES sites (Cosmopolitan), some sites (Intermediate), or only a few sites (Sparse). Scale bar indicates the log10 relative abundance (number of reads normalized by number of non*-Prochlorococcus,* non-*Synechococcus* reads) at a given site. See also Figure S8 for the distribution of some OTUs abundant in bioGEOTRACES, but not prevalent in culture

Because all of the cultures are sourced from seawater samples, we wanted to determine how heterotrophs present in cultures were represented in the global oceans. We used the bioGEOTRACES metagenomics dataset, which spans 610 samples over time and at multiple depths in locations throughout the Atlantic and Pacific Oceans [63] to examine the spatial distributions of heterotrophs present in our cyanobacterial cultures (Fig. 5). We obtained reads mapping to the V4 region of the 16S gene, and clustered the ASVs from our culture collection with the global oceans data at 97% similarity (i.e. 97% OTUs) to accommodate differences in the nature of the data and processing (see Materials and Methods for details). The heterotrophs that dominate global ocean datasets (primarily belonging to the Pelagibacteraceae within the Pelagibacterales) are not well represented in our cultures (Fig. 5b, Figure S8). This absence is not surprising as simply culturing oligotrophs like SAR11 is extremely challenging [64–66]; thus, we expected that the high nutrient concentrations and periodic dilution of cells from culture transfers would enrich for opportunistic copiotrophic organisms.

We next asked how well the prevalent heterotrophic OTUs in cultures were represented in the bioGEOTRACES database. While a few OTUs were readily detected at most sites (Fig. 5c, Figure S8), this was not the general tendency; most of the heterotrophic OTUs that were prevalent in the cultures were rare or sparsely detectable in the wild (Fig. 5c). Of the OTUs that dominated in culture, OTUs that were cosmopolitan across bioGEOTRACES sites included those classified as *Maricaulis*, Alteromonadaceae, and Rhodobacteraceae, while unclassified Gammaproteobacteria, *Alcanivorax*, and *Marinobacter* were intermediately distributed OTUs present in several sites, and OTUs in the genera *Muricauda*, *Thalassospira*, and *Hyphomonas* were generally sparse or absent across the bioGEOTRACES sites (Fig. 5c).

The sparsity of these heterotrophs in the global oceans coupled with their prevalence in cultures of *Prochlorococcus* and *Synechococcus* suggests that they are selected for by the rich culture conditions – conditions that must be patchily distributed in the oceans. As an example, the genus *Muricauda* (family Flavobacteriaceae), which had an OTU well-represented in cultures, but sparsely detectable in bioGEOTRACES (Fig. 5c), is known to be particle-associated, potentially specializing in degradation of high molecular weight organic compounds [67]. Across all cultures in this study, more heterotrophic OTUs in culture were shared with bioGEOTRACES samples taken below the epipelagic zone (> 200 m) than above (0.64% of OTUs below versus 0.3% above, Fisher Exact Test *p*-value = 1e-4). The increase in OTUs shared with cultures at depth might be because of the reliance of heterotrophs on organic carbon as a source of energy in both systems: light energy for photoheterotrophy is unavailable at depth and likely restricted by algae in cultures.

Given the cosmopolitan distribution of some of the prevalent culture heterotrophic OTUs across bioGEOTRACES sites, we expected that they might be positively correlated with picocyanobacterial abundance along the transects. Indeed, we find that across the bioGEOTRACES sites, there is a strong relationship (Spearman’s rho = 0.302, *p*-value = 0) between the abundance of the Rhodobacteraceae OTU, which is ubiquitous in cultures, and the combined abundance of *Prochlorococcus* and *Synechococcus*. Notably, none of the other prevalent culture OTUs showed this relationship, suggesting that this OTU in particular may be coupled to the dynamics of these cyanobacteria in oceans (Table S4). Further laboratory investigations of the interactions of these ubiquitous bacteria with marine picocyanobacteria should reveal interesting and relevant exchanges of matter and energy in the global oceans.

## Conclusions

Although the culture collection being analyzed here was not designed with this study in mind, there are some generalizations we can extract from the heterotrophic “bycatch” that was selected for in the enrichment cultures, which may help guide experiments designed to unravel the co-dependencies in these micro-communities. First, as expected from their obligate oligotrophy, the heterotrophs that numerically dominate the open ocean habitats are not present in these xenic cultures. For most of them, including the abundant SAR11 group, high organic carbon conditions present an obstacle [64, 66] and hence developing a defined medium for their growth was a challenge [65]. Although we previously designed a medium that sustains co-cultures of SAR11 and *Prochlorococcus* in log phase [5], SAR11 dies precipitously when *Prochlorococcus* enters stationary phase, suggesting that strict oligotrophs may not be able to tolerate the accumulation of substrates that occur during *Prochlorococcus* growth – a feature that would have eliminated them in the initial stages of isolating the cyanobacterial strains in our culture collection. This challenge during stationary phase might derive from deleterious effects of high nutrient concentrations on streamlined cellular physiology, with oligotrophs unable to regulate transport rates in the face of high nutrient concentrations [68].

The absence of oligotrophs in the enrichment cultures is in striking contrast to the presence of the numerous copiotrophic heterotrophic strains that thrive in these cultures (this study, and [26–28, 30, 33]) and even “bloom” when *Prochlorococcus* reaches stationary phase [5]. Studies of heterotroph community dynamics in cultures over the course of the exponential and sustained stationary-phase growth [42] have revealed shifts in heterotrophic abundances according to their differential capacities to utilize high- and low-molecular weight dissolved organic compounds. Further work as in that study (using a combination of transcriptomic, metagenomic, and metabolomic approaches) will enable detailed linkage between functional genes in heterotrophic bacteria for metabolism of cyanobacteria-derived photosynthate and the dynamics of these functions in the global oceans.

Interestingly, most of the heterotrophic strains that are widespread among the cultures are not very abundant in the wild. These copiotrophs appear to thrive on high concentrations of organic compounds experienced in culture – an environment that must be patchily distributed in the open ocean habitat [15]. While it is likely that individual phytoplankton can selectively permit the growth of particular heterotrophic bacteria, the sharing of over 40% of OTUs between our dataset and each of the other phytoplankton datasets explored here suggests that phytoplankton may modify their environments in similar ways that lead to conservation of heterotrophic bacterial groups across cultures. It is possible that ubiquitous heterotrophic bacteria in cultures are similar to “broad-range taxa” that process simple metabolic intermediates [67], and thus grow on compounds that are released as generic byproducts of phytoplankton growth. Indeed, such compounds are likely abundant on nutrient rich particles, or ephemeral patches of high organic carbon in the phycosphere of larger phytoplankton [14, 15, 69].

Clearly, to understand the metabolic exchanges between picocyanobacteria and oligotrophic heterotrophs we will have to isolate sympatric strains from the same location. In contrast to standard enrichment approaches, which appear to favor the growth of fast-growing, phycosphere-enriched heterotrophic bacteria, using dilution-to-extinction techniques and low nutrient media is likely to yield more of the abundant, free-living bacteria characteristic of the oligotrophic oceans. Like all challenges with these microorganisms, it is only a matter of time and effort.

## Materials and methods

### Culture maintenance

Previously isolated *Synechococcus* and *Prochlorococcus* from sites spanning the global oceans (Table S1) were maintained by serial passage in natural seawater medium amended with Pro99 nutrients (800 uM N, 50 uM P, trace metals, no added carbon) under low light intensity (~ 10 μmol photons m^− 2^ s^− 1^) in continuous light or a 13:11 light:dark incubator (Moore et al. 2007 [11]). Prior to DNA extractions, cultures were grown until mid-exponential growth phase to increase the comparability of the communities across all cultures.

### Describing the cyanobacteria-associated microbial community composition

We used amplicon sequence variants (hereafter ASVs) of 16S rDNA as our marker. We analyzed the composition of the community of heterotrophs in our isolates when harvested in mid-exponential growth. To this end, we inoculated maintenance cultures (*n* = 74) into fresh medium and allowed them to grow until mid-exponential growth before collecting cells by centrifugation for preparation of 16S rDNA libraries. Sequence libraries had an average depth of 113,602 (min: 72, 369) reads after quality filtering, and in most samples, reads derived predominantly from the cyanobacterial host (*Prochlorococcus* or *Synechococcus*). For the purposes of characterizing the associated community, we excluded reads derived from *Prochlorococcus* or *Synechococcus* in downstream analyses. The cultures contained a median of 11 ASVs (range 1–23) that were at or above an abundance of 0.2%, and a median of 16 ASVs estimated by rarefaction (range 3–31) (Fig. 1b). We do not make quantitative claims about the abundances of ASVs in samples because of the dependence of heterotroph abundance on cyanobacterial growth state [5, 41] and biases in amplicon sequence data [70]; we instead focus on the distribution of observed ASVs, and higher order taxonomic classifications across the cultures.

### Sequence generation and processing

DNA samples were prepared by pelleting 5 mL of exponentially growing cultures by centrifugation at 8000 x g for 15 min. DNA was extracted using the DNeasy Blood & Tissue Kit (QIAGEN Cat No. 69504) following manufacturer instructions. The V4-V5 region of the 16S rRNA gene was amplified using the 515F-Y (5′-GTGYCAGCMGCCGCGGTAA-3′) and 926R (5′-CCGYCAATTYMTTTRAGTT-3′) primers [71], and sequencing libraries prepared in duplicate before pooling using a two-step protocol described previously [72], with the exception of the MED4, NATL2A, and MIT9313 cultures from 2018, which used the same two-step protocol, but were generated in a different sequencing run using 515F (5′-GTGCCAGCMGCCGCGGTAA-3′) and 806R (5′-GGACTACHVGGGTWTCTAAT-3′) primers [72]. The libraries were sequenced on an Illumina MiSeq (Illumina, San Diego, CA, USA) platform, using 250 bp paired-end reads (except for the 2018 MED4, NATL2A, and MIT9313 cultures, which used 150 bp paired-end reads). The fastq sequence data files were processed to generate a table of amplicon sequence variants (ASVs) using DADA2 v1.16 in R [73], with the parameters for each dataset specified in the corresponding R script (https://github.com/microbetrainer/Heterotrophs).

### Mock community construction for sequencing validation

We extracted DNA as described previously from 11 bacteria with distinct V4 16S regions: axenic cultures of *Prochlorococcus* strains MED4, NATL2A, MIT9313, and MIT9312; *Synechococcus* strains WH7803 and WH8102; *Polaribacter* MED152, *Alteromonas macleodii* MIT1002, and three other heterotrophic bacteria. We amplified the full 16S gene using the primers 27F (5′-AGAGTTTGATCMTGGCTCAG-3′) and 1492R (5′-GGTTACCTTGTTACGACTT-3′), verified the 16S sequence by Sanger sequencing (Eton Bioscience, Inc. San Diego, CA, USA), then cloned the 16S gene from each culture using the Zero Blunt™ TOPO™ PCR Cloning Kit (ThermoFisher Cat #450245). The resulting plasmids were combined either in equimolar concentrations or in a two-fold dilution series (with the most concentrated 16S 1024x more concentrated than the least) with three technical replicates, which were included as samples in 16S DNA library preparation.

### 16S amplicon sequence data analysis

In order to limit the extent to which we were measuring contaminating PCR products from adjacent wells, we excluded ASVs that had anomalously low abundance in one well given its abundance in an adjacent well. Because this approach is conservative (and likely removing true positive ASVs), it will have the tendency to deflate the estimates of taxonomic richness within a sample. We applied the following filter: if the relative abundance of an ASV in a specified well was less than 10% that of the maximum relative abundance in an adjacent well on the 96-well plate, the relative abundance of the ASV in the specified well was set to 0. Based on analysis of the mock community data, we found no contaminating sequences at a relative abundance of more than 0.2%, thus, sequences were excluded from a sample if they had a relative abundance lower than 0.2%. This cutoff tends to be conservative, as rarefaction analysis retained more ASVs per sample overall. For all analyses not involving OTU clustering, the sequences were trimmed to the same length. The sequences were imported to QIIME 2 v2019.10.0 [74], where they were dereplicated using VSEARCH v2.7.0 [75] and assigned taxonomies using the gg-13-8-99-nb-classifier [76, 77]. Using fasttree v2.1.10 [78] and mafft alignment v7.3.10 [79] within QIIME 2, phylogeny of the non*-Prochlorococcus,* non-*Synechococcus* ASVs was created. To identify heterotrophs associated with either *Prochlorococcus* or *Synechococcus* cultures, an indicator species analysis was performed: in R, the multipatt function within the indicspecies package v1.7.9 [80] was applied to the presence/absence of heterotrophic ASVs across *Prochlorococcus* and *Synechococcus* cultures. Univariate PERMANOVA was conducted using the adonis2 function within the vegan v2.5–6 package [81] in R, using 1000 permutations each (see Table S5). Interaction effects were tested by each pairwise combination of factors using the by = “margin” option in adonis2. Post-hoc tests on pairwise beta-dispersion were conducted using the pairwise adonis approach in R (https://github.com/pmartinezarbizu/pairwiseAdonis/blob/master/pairwiseAdonis/R/pairwise.adonis.R).

### Comparative analysis of diatom and Synechococcus culture datasets

We compared sequences identified in this study to sequences found in newly isolated *Synechococcus* cultures [41] and sequences isolated from the phycosphere of diatoms in culture [56]. The *Synechococcus* study [41] used 520F (5′-AYTGGGYDTAAAGNG-3′) and 802R (5′-TACNVGGGTATCTAATCC-3′) for 16S data generation and the second study used 515F (5′-GTGYCAGCMGCCGCGGTAA-3′) and 786R (5′-GGACTACNVGGGTWTCTAAT-3′). Both sets of primers produce sequences that are nested inside those generated in this study (515Y-F, 926R). The diatom dataset contained microbial communities measured over time in cultures of four strains of *Asterionellopsis glacialis* and three strains of *Nitzschia longissima*. We included the initial measurements of microbial communities from nine new *Synechococcus* isolates, four from oligotrophic waters and five from eutrophic waters. For this analysis, we clustered at 97% identity (hereafter, OTUs) due to differences in the generation of the amplicon data in the published studies. The sequences from both studies were processed in DADA2 [73] to produce a table of ASVs, with the parameters specified in the corresponding R script (https://github.com/microbetrainer/Heterotrophs). We did not trim ASVs from these three datasets to the same length. We excluded ASVs that were identified as either mitochondria or chloroplast from analysis, and excluded ASVs from a sample if they had a relative abundance below 0.2%. In QIIME 2 [74], the ASVs were clustered at 97% identity using VSEARCH [75], and the taxonomy of each cluster was assigned using the gg-13-8-99-nb-classifier [76, 77]. The samples were clustered using Ward’s method of hierarchical clustering on the unweighted UniFrac distance at the 97% OTU level [82] calculated with the phyloseq package v1.28.0 in R [83]. NMDS plots were generated using the adonis2 function on the unweighted UniFrac distance in the R phyloseq package [83].

### BioGEOTRACES comparisons

PhyloFlash v3.0 (https://github.com/HRGV/phyloFlash) [84] was used to obtain SSU rRNA reads mapping to the 16S genes in bioGEOTRACES metagenomes [63] and low complexity reads were removed using komplexity v0.3.6 (https://github.com/eclarke/komplexity). Bacterial reads were obtained using bbsplit v38.22 (https://sourceforge.net/projects/bbmap/) with curated databases (https://osf.io/e65rs/) from SILVA 132 and aligned to group-specific references using PyNAST v1.2.2 [85]. Custom scripts were then used to obtain metagenomic reads mapping to the primer region for the 515Y/926R primer pair, and were followed by low-complexity masking and quality filtering all courtesy of Jesse McNichol (data and code can be found at https://osf.io/n5ftw/, and the full workflow at https://github.com/jcmcnch/MGPrimerEval). The final fastqs containing metagenomic reads mapping to the 515Y/926R region were used as input for downstream analysis. These bioGEOTRACES reads and the culture ASVs were imported into QIIME 2 [74] through which the sequences were dereplicated and clustered at 97% identity with VSEARCH [75]. The OTUs were then assigned taxonomies using the gg-13-8-99-nb-classifier [76, 77] and represented on maps using the accompanying bioGEOTRACES metadata.

### Comparison of 2018 and 2019 heterotroph communities

The cultures described in this study were sampled in the summer of 2019. The non-axenic cultures of *Prochlorococcus* MED4, MIT9313, and NATL2A were sampled in 2018 and again in 2019. To investigate whether the composition of heterotrophs changed over this period, we compared the ASVs in these cultures sampled over time. Using DADA2 [73], we trimmed the primers (the first 20 bases) from the reads from the 2018 samples, as quality decreased at this point in the sequences. We trimmed the ASVs from the two sources to the same length. For both datasets, ASVs were excluded from a sample if they had a relative abundance less than 0.2%. In QIIME 2 [74], the ASVs were dereplicated across the two datasets using VSEARCH [75] and assigned taxonomies using the gg-13-8-99-nb-classifier [76, 77]. Within QIIME 2 [74], fasttree [78], and mafft [79] were used to create a phylogeny of the non*-Prochlorococcus*, non*-Synechococcus* ASVs. The abundance of each ASV in each sample was normalized by the number of *Prochlorococcus* sequences in the sample. The Spearman correlation between each pair of samples was calculated based on the abundance of each non-*Prochlorococcus* ASV normalized by the number of *Prochlorococcus* reads.

### Accession numbers

The 16S rDNA sequence data from this study were deposited in the NCBI Sequence Read Archive under BioProject ID PRJNA607777.

## Supplementary Information

**Additional file 1: Table S1.** Metadata associated with the isolation of each cyanobacterial culture. **Table S2.** Indicator Species associated with either Prochlorococcus or Synechococcus cultures. **Table S3.** OTUs shared between this study, Behringer et al. 2018 [55], and Zheng et al. 2018 [40] datasets. **Table S4.** Spearman correlation between OTUs in Fig. 5c and aggregate abundance of Prochlorococcus and Synechococcus in bioGEOTRACES. **Table S5.** Results of PERMANOVA Analysis using adonis2 with univariate tests of main effects and tests for interaction effects of pairwise combinations of factors.

**Additional file 2: Figure S1.** Stability of heterotroph community composition in three cultures sampled 1 year apart. (A) The *Prochlorococcus*-normalized relative abundance (indicated by the color bar) of each of the heterotroph ASVs is shown in the heatmap for each of three cyanobacterial enrichment cultures (*Prochlorococcus* strains MED4, MIT9313, and NATL2A) sampled once in 2018 and again in 2019 for amplicon sequencing. The phylogenetic tree on the left is based on the heterotroph ASV sequences. (B) The Spearman correlation (comparing the correspondence in rank) of relative abundance of heterotroph ASVs is indicated by the color bar. **Figure S2.** Composition of heterotroph communities, as defined by class membership of ASVs, in long-term cultures of *Prochlorococcus* (green) and *Synechococcus* (magenta) hosts. This is the same data as depicted in Fig. 2, but here the heterotroph communities in each culture are arranged using Ward’s method of hierarchical clustering with unweighted UniFrac on ASVs as the distance metric to emphasize the relationships between communities. Relative abundance of heterotrophic community members, by class, is shown to the right of each strain name. **Figure S3.** Mapping of cultures onto ordination plots labeled to indicate the position of each cyanobacterial host’s community at the ASV level in the ordination plots from Fig. 4 and Figure S4. Non-metric multidimensional scaling (NMDS) of heterotroph communities using unweighted UniFrac as the distance metric. Each point in the NMDS plot represents a single heterotroph community associated with the given cyanobacterial host indicated in the name. The closeness of two points in NMDS space reflects the distance between communities, with communities having more similar phylogenetic structure (as measured by unweighted UniFrac) grouping more closely together. **Figure S4.** Ordination of heterotroph community composition at the ASV level overlaid with enrichment culture metadata. Non-metric multidimensional scaling (NMDS) of heterotroph communities using unweighted UniFrac as the distance metric overlaid with (A) cyanobacterial ecotype, (B) isolation location, (C) cyanobacterial clade, and (D) culture age. The closeness of two points in NMDS space reflects the distance between communities, with communities having more similar phylogenetic structure (as measured by unweighted UniFrac) grouping more closely together. Heterotroph communities for which metadata was not known are indicated as NAs. See also Fig. 4, and Figures S3 and S7. **Figure S5.** Venn diagram showing the number of OTUs shared in heterotroph communities from diatoms (Behringer et al. 2018 [55]) and *Synechococcus* (Zheng et al. 2018 [40]) compared to the *Prochlorococcus* and *Synechococcus* in this study. The area of the ellipses is not to scale with the number of OTUs. **Figure S6** Heterotroph communities accompanying diatoms (dark blue) (Behringer et al. 2018 [55]) and *Synechococcus* (orange) in culture for less than a year (Zheng et al. 2018 [40]) compared to this study (green (*Prochlorococcus*) and magenta (*Synechococcus*)) with cultures over 5 years old. Bacterial classes are indicated by the colors in the legend, and relative abundance of each class in the heterotroph community of the corresponding culture is shown. Heterotroph communities in each cyanobacterial culture (vertical axis) are organized by the hierarchical clustering tree on the left using Ward’s method of hierarchical clustering with unweighted UniFrac as the distance metric on 97% OTUs. See also Fig. 2 and Figure S2. **Figure S7** Associations between metadata pertaining to isolation or host phylogeny of enrichment cultures. The pairwise association between each variable indicated on the vertical and horizontal axes were calculated using an asymmetric measure of association, Goodman and Kruskal’s τ, ranging from 0 to 1. A τ of 1 indicates that there is a perfect correspondence between the levels in one variable and the record index. For instance, the clade has a τ of 1 with ecotype because clades are perfectly nested within ecotypes (that is, identification of the clade is sufficient to identify the ecotype). Because of the asymmetry in the metric, ecotype has a τ less than 1, but greater than 0 with the clade; ecotype only constrains identification of clades, but does not perfectly determine them. See also Figs. 4, S3, and S4. **Figure S8.** (A) Top two most abundant OTUs in bioGEOTRACES; these OTUs are not present in cyanobacterial cultures. (B) Top two most abundant OTUs (distinct unclassified OTUs in the family Rhodoobacteraceae) in bioGEOTRACES that are also present in the cyanobacterial cultures. Scale bar indicates the log10 relative abundance (number of reads normalized by number of non*-Prochlorococcus,* non-*Synechococcus* reads) at a given site. Related to Fig. 5.

## Data Availability

The datasets generated and/or analyzed during the current study are available through NCBI Sequence Read Archive under BioProject ID PRJNA607777. All other data are included in this published article.
