# Supplementary material for: Microbial diversity of co-occurring heterotrophs in cultures of marine picocyanobacteria
Source: Environ Microbiome. 2021 Jan 6;16:1. doi: 10.1186/s40793-020-00370-x (PMC8067657; doi:10.1186/s40793-020-00370-x)
Supplement: Supplementary file 2 — Additional file 2: Figure S1. Stability of heterotroph community composition in three cultures sampled 1 year apart. (A) The Prochlorococcus-normalized relative abundance (indicated by the color bar) of each of the heterotroph ASVs is shown in the heatmap for each of three cyanobacterial enrichment cultures (Prochlorococcus strains MED4, MIT9313, and NATL2A) sampled once in 2018 and again in 2019 for amplicon sequencing. The phylogenetic tree on the left is based on the heterotroph ASV sequences. (B) The Spearman correlation (comparing the correspondence in rank) of relative abundance of heterotroph ASVs is indicated by the color bar. Figure S2. Composition of heterotroph communities, as defined by class membership of ASVs, in long-term cultures of Prochlorococcus (green) and Synechococcus (magenta) hosts. This is the same data as depicted in Fig. 2, but here the heterotroph communities in each culture are arranged using Ward’s method of hierarchical clustering with unweighted UniFrac on ASVs as the distance metric to emphasize the relationships between communities. Relative abundance of heterotrophic community members, by class, is shown to the right of each strain name. Figure S3. Mapping of cultures onto ordination plots labeled to indicate the position of each cyanobacterial host’s community at the ASV level in the ordination plots from Fig. 4 and Figure S4. Non-metric multidimensional scaling (NMDS) of heterotroph communities using unweighted UniFrac as the distance metric. Each point in the NMDS plot represents a single heterotroph community associated with the given cyanobacterial host indicated in the name. The closeness of two points in NMDS space reflects the distance between communities, with communities having more similar phylogenetic structure (as measured by unweighted UniFrac) grouping more closely together. Figure S4. Ordination of heterotroph community composition at the ASV level overlaid with enrichment culture metadata. Non-metric multidimension [file 40793_2020_370_MOESM2_ESM.docx]

**SUPPLEMENTARY FIGURES**

**
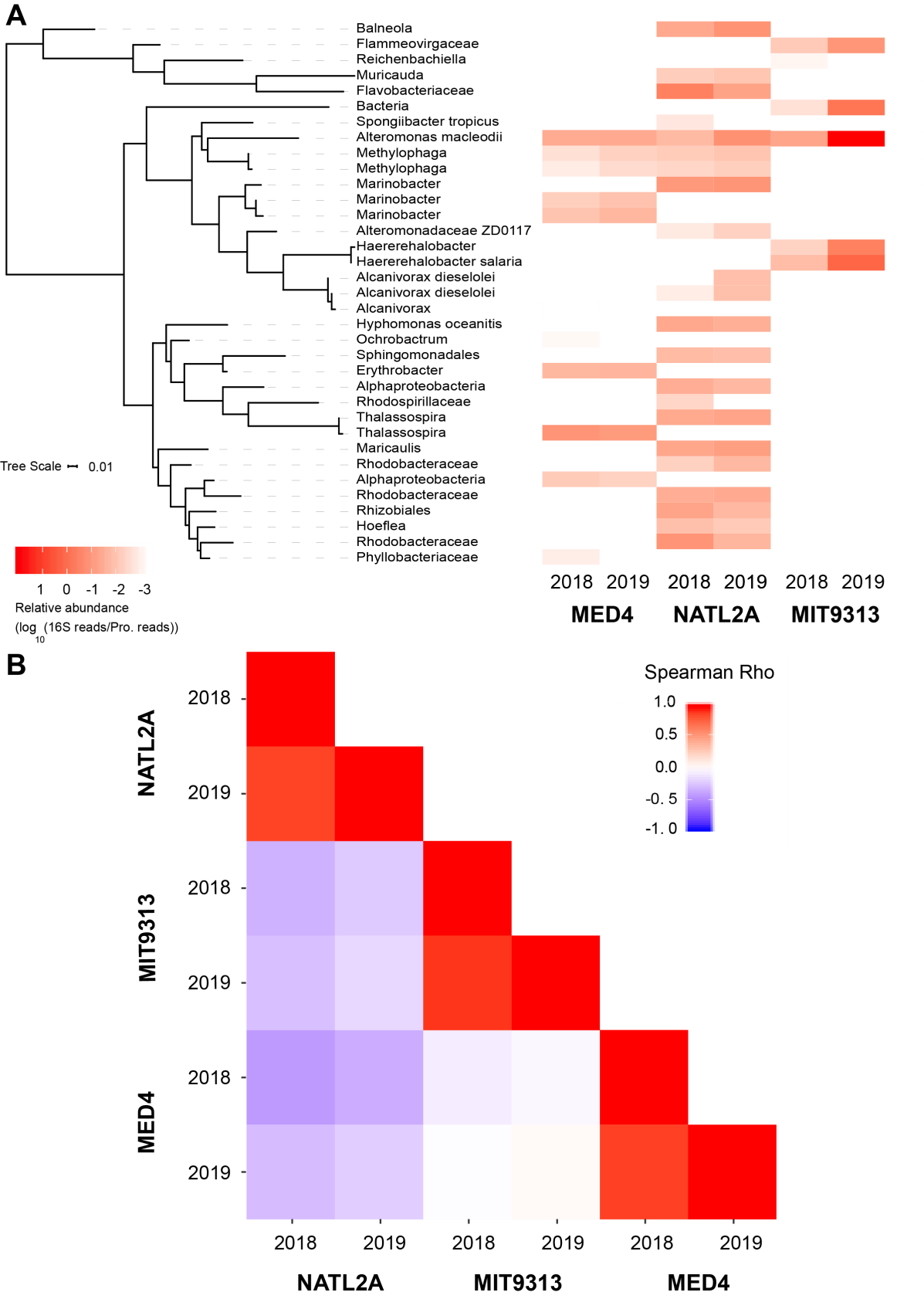
**

**Figure S1.** Stability of heterotroph community composition in three cultures sampled one year apart. (A) The *Prochlorococcus*-normalized relative abundance (indicated by the color bar) of each of the heterotroph ASVs is shown in the heatmap for each of three cyanobacterial enrichment cultures (*Prochlorococcus* strains MED4, MIT9313, and NATL2A) sampled once in 2018 and again in 2019 for amplicon sequencing. The phylogenetic tree on the left is based on the heterotroph ASV sequences. (B) The Spearman correlation (comparing the correspondence in rank) of relative abundance of heterotroph ASVs is indicated by the color bar.


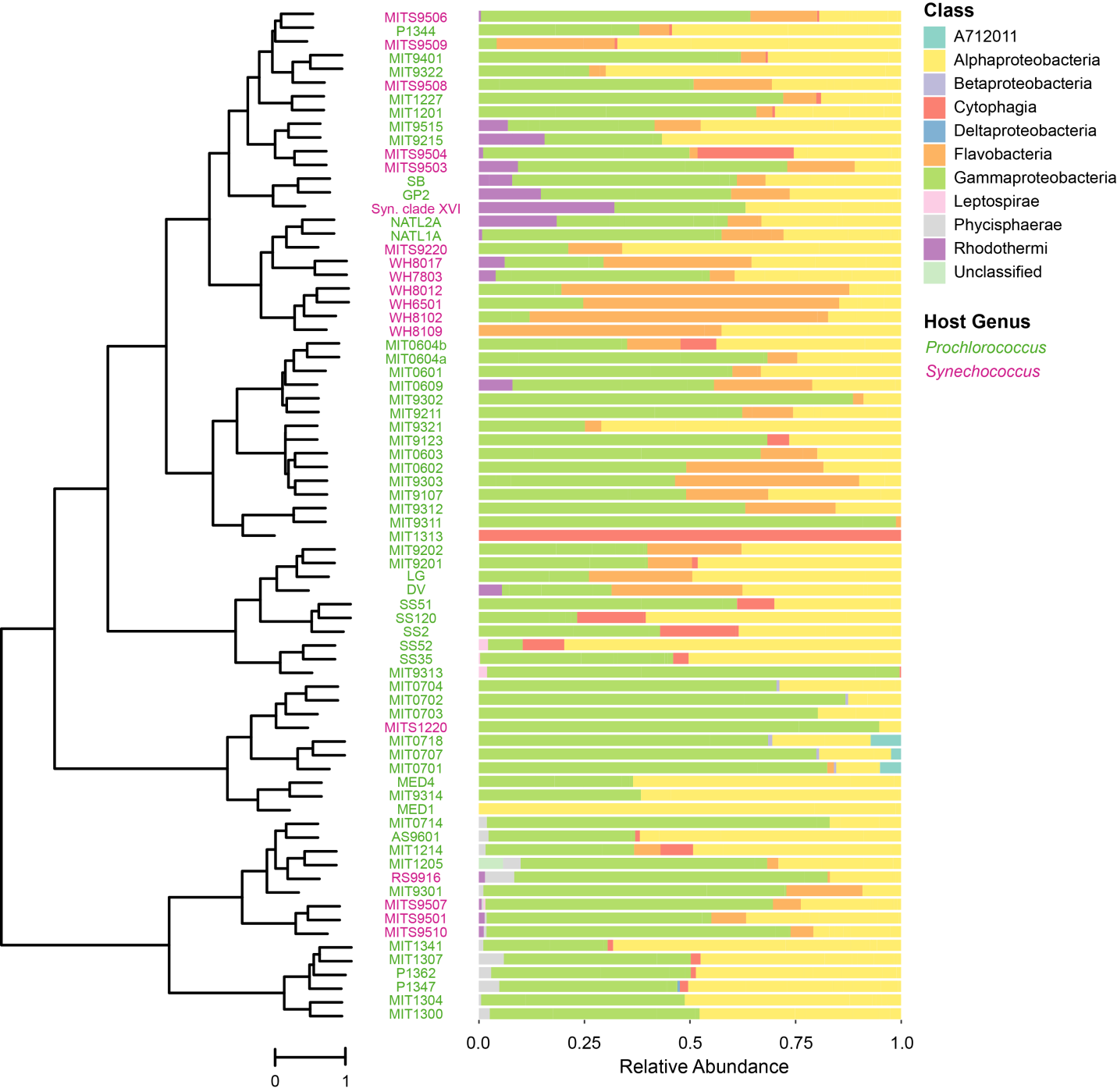


**Figure S2.** Composition of heterotroph communities, as defined by class membership of ASVs, in long-term cultures of *Prochlorococcus* (green) and *Synechococcus* (magenta) hosts. This is the same data as depicted in Fig. 2, but here the heterotroph communities in each culture are arranged using Ward’s method of hierarchical clustering with unweighted UniFrac on ASVs as the distance metric to emphasize the relationships between communities. Relative abundance of heterotrophic community members, by class, is shown to the right of each strain name.

**
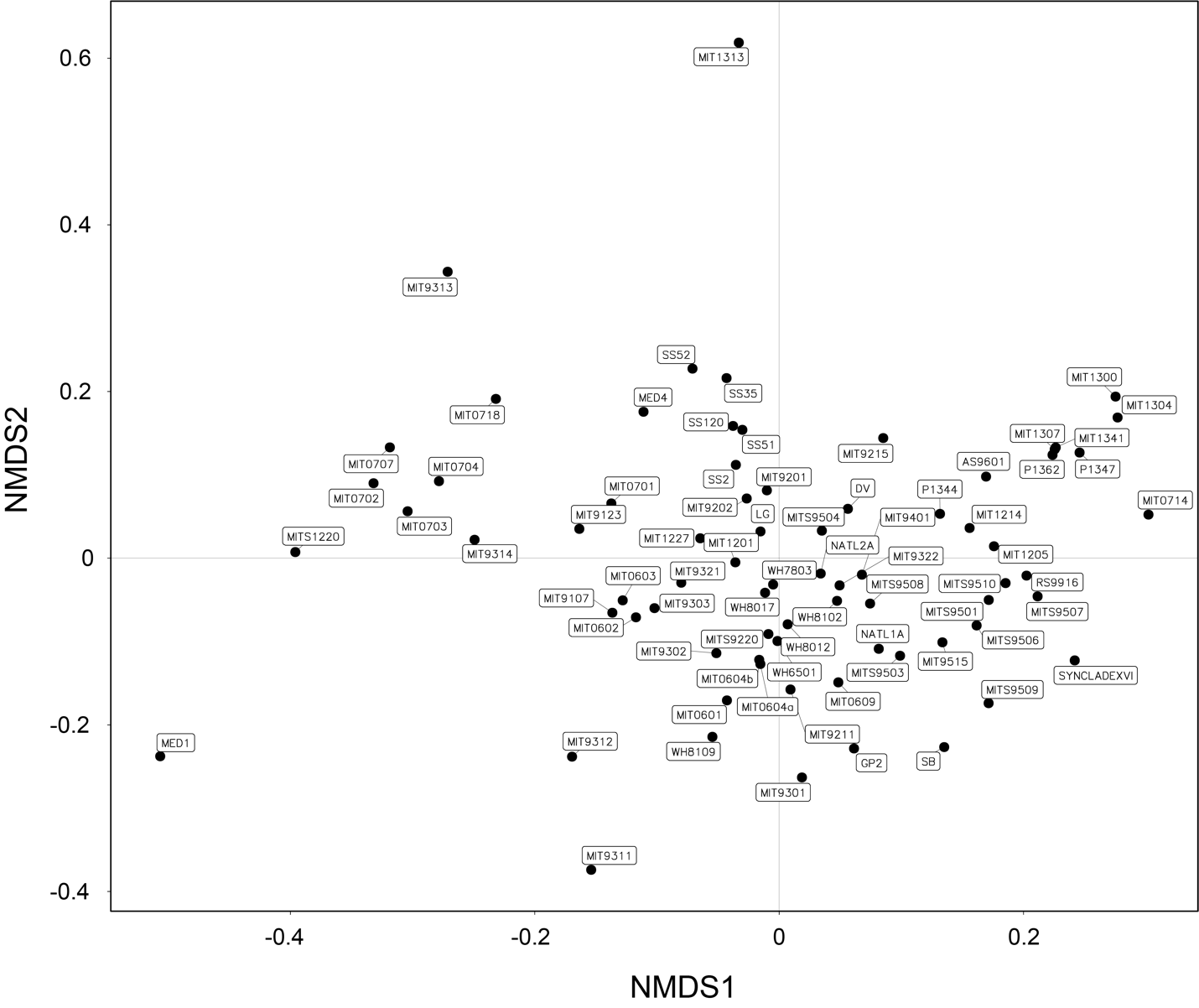
**

**Figure S3.** Mapping of cultures onto ordination plots labeled to indicate the position of each cyanobacterial host’s community at the ASV level in the ordination plots from Figure 4 and Figure S4. Non-metric multidimensional scaling (NMDS) of heterotroph communities using unweighted UniFrac as the distance metric. Each point in the NMDS plot represents a single heterotroph community associated with the given cyanobacterial host indicated in the name. The closeness of two points in NMDS space reflects the distance between communities, with communities having more similar phylogenetic structure (as measured by unweighted UniFrac) grouping more closely together.

**
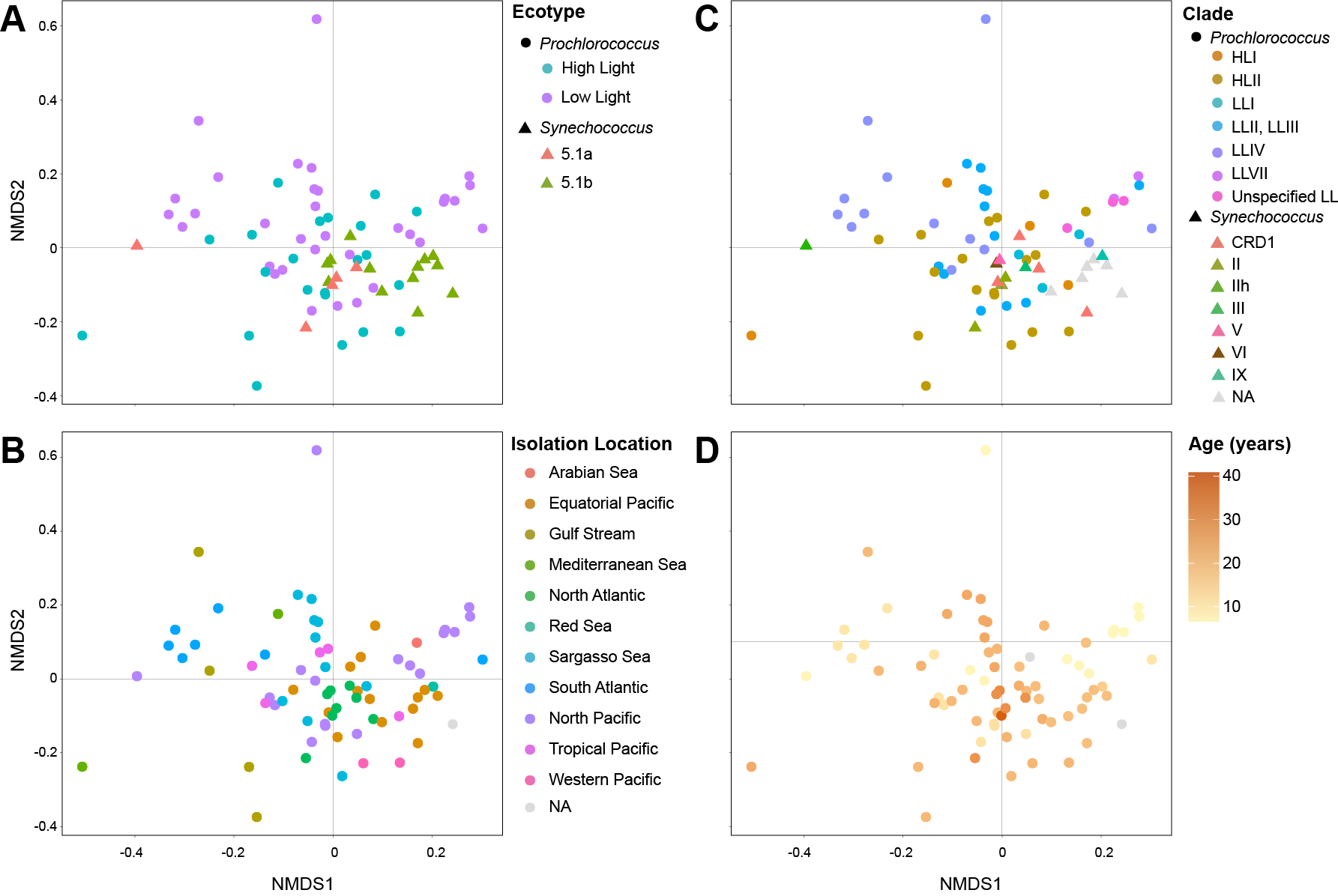
**

**Figure S4.** Ordination of heterotroph community composition at the ASV level overlaid with enrichment culture metadata. Non-metric multidimensional scaling (NMDS) of heterotroph communities using unweighted UniFrac as the distance metric overlaid with (A) cyanobacterial ecotype, (B) isolation location, (C) cyanobacterial clade, and (D) culture age. The closeness of two points in NMDS space reflects the distance between communities, with communities having more similar phylogenetic structure (as measured by unweighted UniFrac) grouping more closely together. Heterotroph communities for which metadata was not known are indicated as NAs. See also Figure 4, and Figures S3 and S7.

**
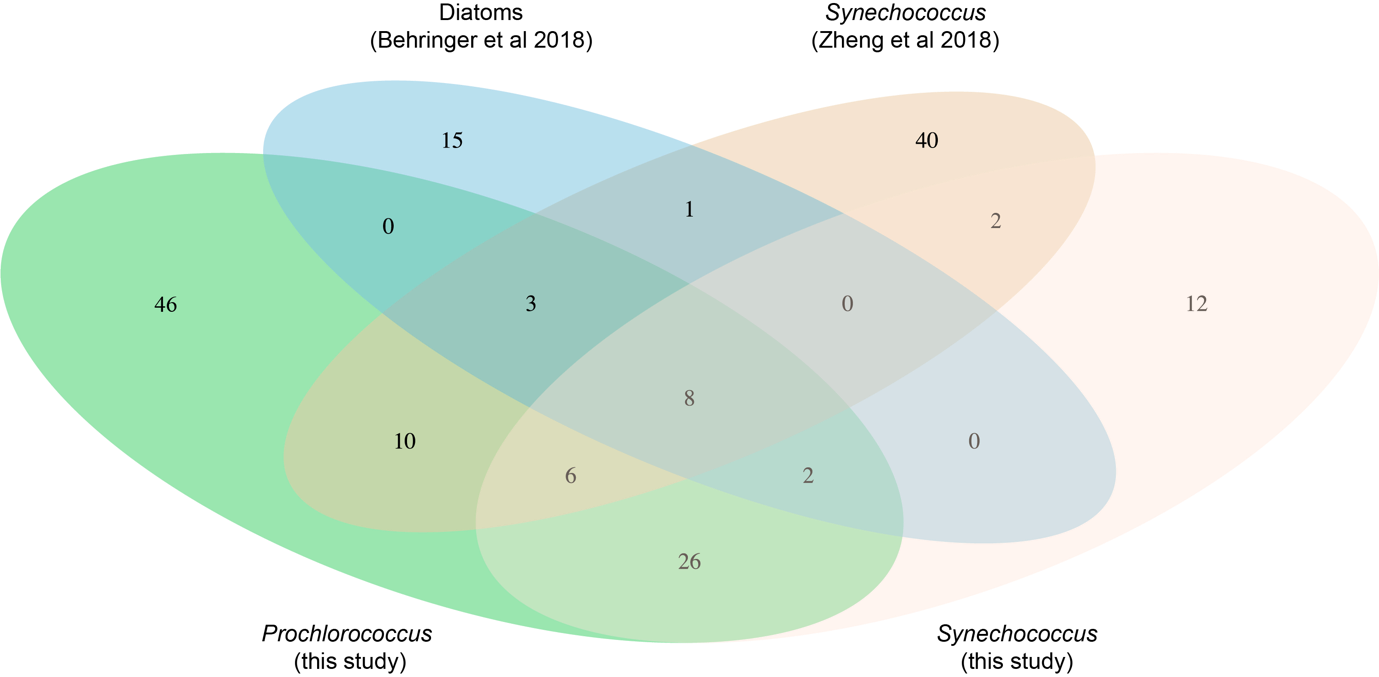
**

**Figure S5.** Venn diagram showing the number of OTUs shared in heterotroph communities from diatoms (Behringer et al., 2018) and *Synechococcus* (Zheng et al., 2018) compared to the *Prochlorococcus* and *Synechococcus* in this study. The area of the ellipses is not to scale with the number of OTUs.

**
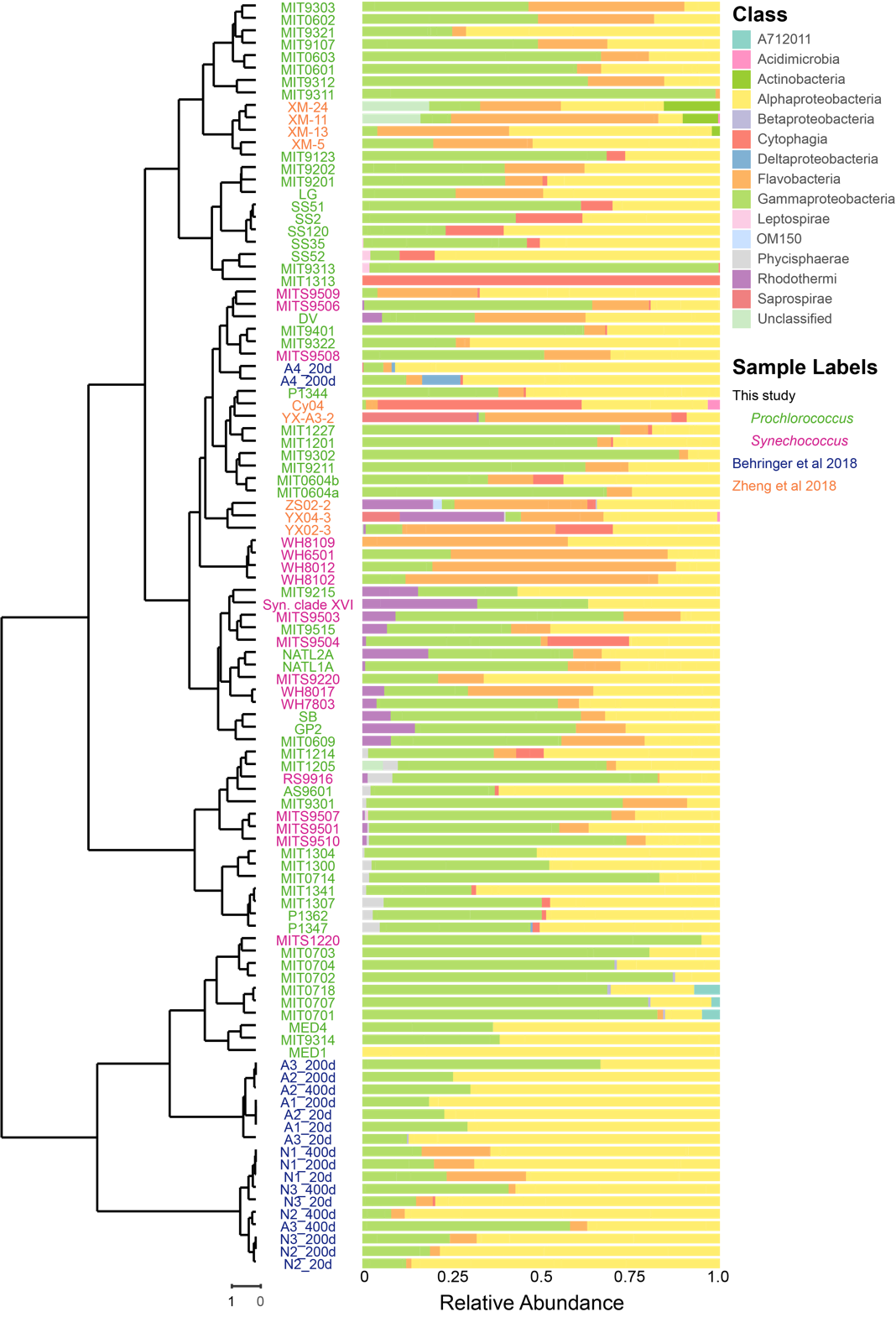
**

**Figure S6** Heterotroph communities accompanying diatoms (dark blue) (Behringer et al., 2018) and *Synechococcus* (orange) in culture for less than a year (Zheng et al., 2018) compared to this study (green (*Prochlorococcus*) and magenta (*Synechococcus*)) with cultures over 5 years old. Bacterial classes are indicated by the colors in the legend, and relative abundance of each class in the heterotroph community of the corresponding culture is shown. Heterotroph communities in each cyanobacterial culture (vertical axis) are organized by the hierarchical clustering tree on the left using Ward’s method of hierarchical clustering with unweighted UniFrac as the distance metric on 97% OTUs. See also Figure 2 and Figure S2.

**
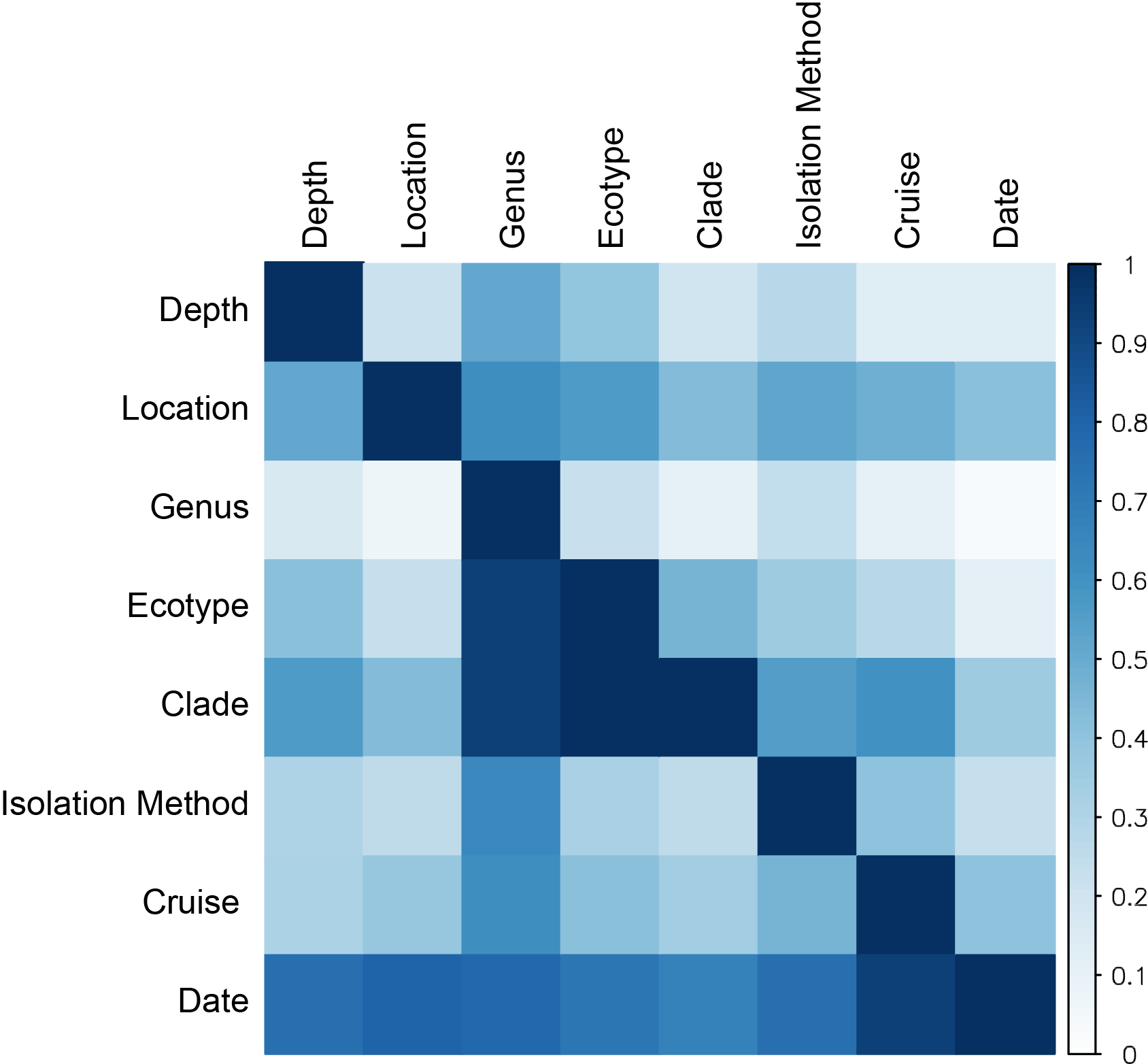
**

**Figure S7** Associations between metadata pertaining to isolation or host phylogeny of enrichment cultures. The pairwise association between each variable indicated on the vertical and horizontal axes were calculated using an asymmetric measure of association, Goodman and Kruskal’s τ, ranging from 0 to 1. A τ of 1 indicates that there is a perfect correspondence between the levels in one variable and the record index. For instance, the clade has a τ of 1 with ecotype because clades are perfectly nested within ecotypes (that is, identification of the clade is sufficient to identify the ecotype). Because of the asymmetry in the metric, ecotype has a τ less than 1, but greater than 0 with the clade; ecotype only constrains identification of clades, but does not perfectly determine them.

See also Figures 4, S3, and S4.

**
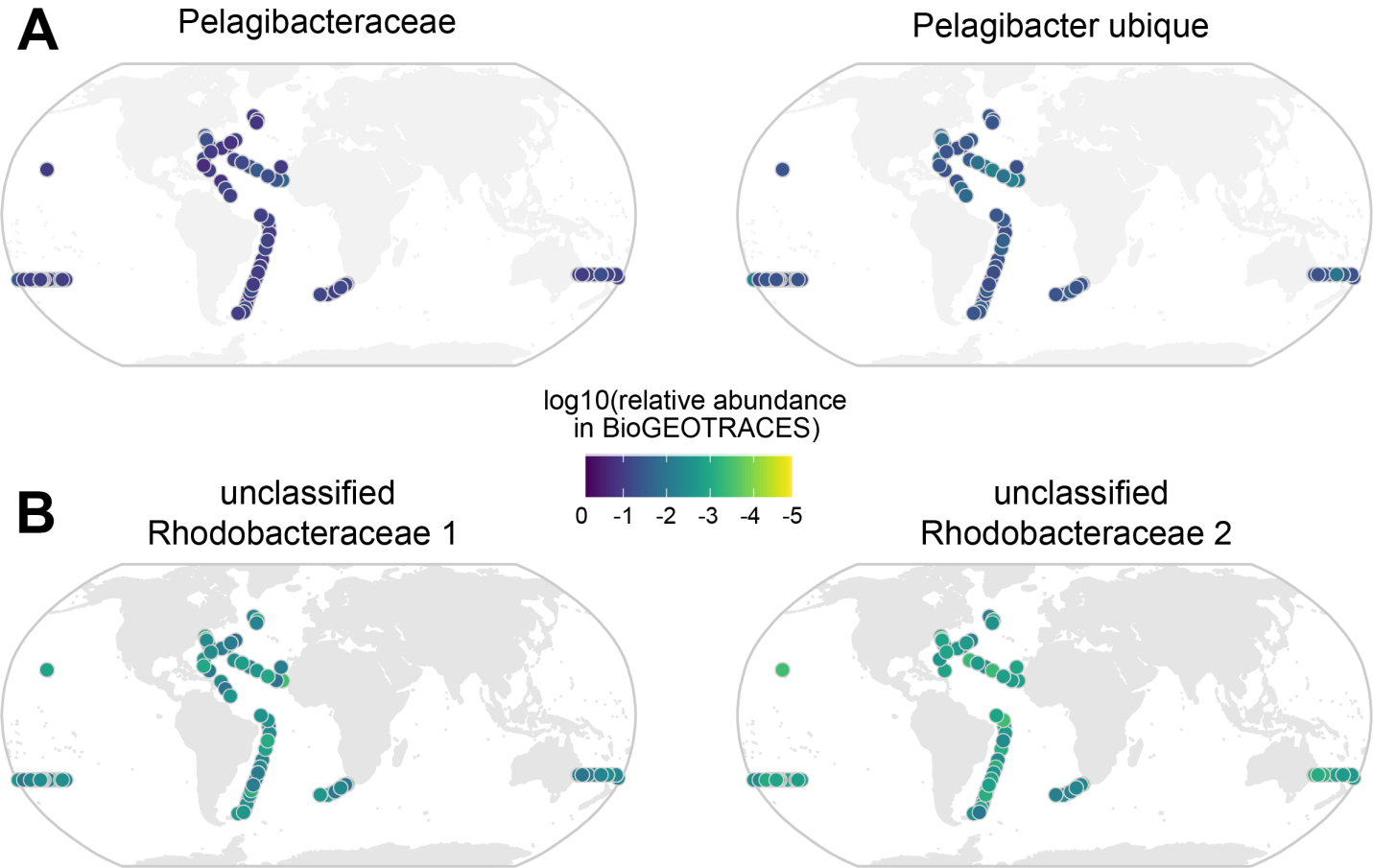
**

**Figure S8.** (A) Top two most abundant OTUs in bioGEOTRACES; these OTUs are not present in cyanobacterial cultures. (B) Top two most abundant OTUs (distinct unclassified OTUs in the family Rhodoobacteraceae) in bioGEOTRACES that are also present in the cyanobacterial cultures. Scale bar indicates the log10 relative abundance (number of reads normalized by number of non*-Prochlorococcus,* non-*Synechococcus* reads) at a given site. Related to Figure 5.
